# Impact of General Anesthesia Guided by State Entropy (SE) and Response Entropy (RE) on Perioperative Stability in Elective Laparoscopic Cholecystectomy Patients—A Prospective Observational Randomized Monocentric Study

**DOI:** 10.3390/e22030356

**Published:** 2020-03-19

**Authors:** Anca Raluca Dinu, Alexandru Florin Rogobete, Sonia Elena Popovici, Ovidiu Horea Bedreag, Marius Papurica, Corina Maria Dumbuleu, Raluca Ramona Velovan, Daiana Toma, Corina Maria Georgescu, Lavinia Ioana Trache, Claudiu Barsac, Loredana Luca, Bettina Buzzi, Andra Maghiar, Mihai Alexandru Sandesc, Samir Rimawi, Madalin Marian Vaduva, Lavinia Melania Bratu, Paul Manuel Luminosu, Dorel Sandesc

**Affiliations:** 1 Faculty of Medicine, “Victor Babes” University of Medicine and Pharmacy, Timisoara 300041, Romania; anca.dinu.umft@gmail.com (A.R.D.); bedreag.ovidiu@umft.ro (O.H.B.); marius.papurica@gmail.com (M.P.); lavi.ceuta@gmail.com (L.M.B.); dsandescumft@gmail.com (D.S.); 2 Clinic of Anaesthesia and Intensive Care, Emergency County Hospital “Pius Brinzeu”, Timisoara 325100, Romania; popovici.sonia@yahoo.com (S.E.P.); corinamaria_87@yahoo.com (C.M.D.); raluca_velovann@yahoo.com (R.R.V.); daiana.toma@yahoo.com (D.T.); corinageorgescu28.85@yahoo.com (C.M.G.); lavinia_trache@yahoo.com (L.I.T.); claudiu_barsac@yahoo.com (C.B.); loredana_ati@yahoo.com (L.L.); bettynabuzzi@yahoo.com (B.B.); andramaghiar@yahoo.com (A.M.); rimawi.samir@gmail.com (S.R.); vaduvamadalin14@gmail.com (M.M.V.); luminosu_paul@yahoo.com (P.M.L.); 3 Department of Clinical Research and Medical Education, Romanian Society of Anaesthesia and Intensive Care (SRATI), Timisoara 325100, Romania

**Keywords:** state entropy, response entropy, general anesthesia, patient safety, recovery

## Abstract

Laparoscopic cholecystectomy is one of the most frequently performed interventions in general surgery departments. Some of the most important aims in achieving perioperative stability in these patients is diminishing the impact of general anesthesia on the hemodynamic stability and the optimization of anesthetic drug doses based on the individual clinical profile of each patient. The objective of this study is the evaluation of the impact, as monitored through entropy (both state entropy (SE) and response entropy (RE)), that the depth of anesthesia has on the hemodynamic stability, as well as the doses of volatile anesthetic. A prospective, observational, randomized, and monocentric study was carried out between January and December 2019 in the Clinic of Anesthesia and Intensive Care of the “Pius Brînzeu” Emergency County Hospital in Timișoara, Romania. The patients included in the study were divided in two study groups: patients in Group A (target group) received multimodal monitoring, which included monitoring of standard parameters and of entropy (SE and RE); while the patients in Group B (control group) only received standard monitoring. The anesthetic dose in group A was optimized to achieve a target entropy of 40–60. A total of 68 patients met the inclusion criteria and were allocated to one of the two study groups: group A (N = 43) or group B (N = 25). There were no statistically significant differences identified between the two groups for both demographical and clinical characteristics (p > 0.05). Statistically significant differences were identified for the number of hypotensive episodes (p = 0.011, 95% CI: [0.1851, 0.7042]) and for the number of episodes of bradycardia (p < 0.0001, 95% CI: [0.3296, 0.7923]). Moreover, there was a significant difference in the Sevoflurane consumption between the two study groups (p = 0.0498, 95% CI: [−0.3942, 0.9047]). The implementation of the multimodal monitoring protocol, including the standard parameters and the measurement of entropy for determining the depth of anesthesia (SE and RE) led to a considerable improvement in perioperative hemodynamic stability. Furthermore, optimizing the doses of anesthetic drugs based on the individual clinical profile of each patient led to a considerable decrease in drug consumption, as well as to a lower incidence of hemodynamic side-effects.

## 1. Introduction

Globally, laparoscopic cholecystectomy is considered to be the most frequently performed intervention in the field of general and abdominal surgery [1]. In recent years, the incidence of pathological processes of the gall bladder has increased by over 50% in certain regions [1]. Therefore, the number of patients admitted for specific laparoscopic interventions has increased significantly, leading to an increase in the number of post-operative complications. These are reflected both in the clinical evolution, with increased length of stay and decreased patient satisfaction, as well as in the economic segment of healthcare [1,2].

A series of recent guidelines recommend multimodal monitoring of general anesthesia in order to increase patient safety. The minimal mandatory monitoring includes pulse oximetry, electrocardiography, non-invasive monitoring of arterial blood pressure (NIBP), capnography (EtCO_2_), fraction of inspired oxygen (FiO_2_), the fraction of expired oxygen (FeO_2_), anesthetic gas concentration, airway pressure, and temperature. Recent recommendations have focused on the introduction in the daily routine of certain additional parameters, such as degree of hypnosis monitoring (depth of anesthesia), the evaluation of nociception–antinociception balance, and neuromuscular transmission monitoring. Depending on the clinical profile of each patient, advanced monitoring can be further extended by introducing special parameters for the evaluation of hemodynamic status. 

However, a high number of general anesthetics are administered with no advance monitoring, based only on clinical signs such as lacrimation, sweating, changes in heart rate and blood pressure, or major ventilatory imbalances, which could be directly correlated with the degree of hypnosis. Another parameter routinely used, but which has been questioned recently, is the minimum alveolar concentration (MAC). Based on the literature, researchers have concluded that MAC cannot guarantee balanced anesthesia, as it can vary based on different factors such as the type of surgery, patient comorbidities, or age. 

The most common techniques used for monitoring the degree of hypnosis are systems based on EEG-signal interpretation [3,4,5,6]. For a more user-friendly interface in clinical practice, the technology transforms these stages into numerical values in the interval between 0 and 100. Entropy is a technology that integrates two methods: the deciphering of EEG signals and the electromyography (EMG) of face muscles. Entropy, as used in hypnosis monitoring, is a mathematical concept used for the interpretation of non-linear dynamic data that gives two values: state entropy (SE) and response entropy (RE). Technically, SE is characterized by the Shannon entropy [7], the first discovered and studied parameter. RE shows the regularity of frequency distribution in the 0–47 Hz interval and includes both EEG and EMG activity. In contrast, SE only includes EEG analysis and calculates frequencies in the 0–32 Hz interval [8,9]. Even with these values available, there is presently no gold standard for monitoring the degree of hypnosis in general anesthesia. In order to integrate technology in clinical practice, it is preferable to apply the concept of multimodal monitoring, including the monitoring of the degree of hypnosis alongside the classical hemodynamic and respiratory variables.

The main objective of this study is to analyze the statistical and clinical impacts of a multimodal monitoring protocol including classical monitoring parameters as well as depth of anesthesia monitoring through entropy. The secondary objectives are analyzing its impact on drug consumption and analyzing the general clinical prognoses of these patients. 

## 2. Materials and Methods 

### 2.1. Study Population

A prospective, observational, and randomized study was carried out in the Clinic for Anesthesia and Intensive Care of the “Pius Brînzeu” Emergency County Hospital, Timișoara, Romania between January and December 2019. The study is part of a larger group of clinical studies of the Department for Research and Medical Education of the Romanian Society of Anesthesia and Intensive Care in Romania (https://www.srati.ro/). Approval of the Ethical Committee, Emergency University County Hospital “Pius Brinzeu” Timisoara, Romania, ID: ROE20177, Approval of ClinicalTrials.Gov, USA, ID: NCT03210077 and Approval of Ethical Committee, Romanian Society of Anaesthesia and Intensive Care, Romania, ID: ROE20171. This study was approved by the Ethics Committee of the institution and all the procedures respected the Helsinki Declaration for clinical studies and patient safety. 

The included patients were randomized in two study groups: the multimodal monitoring protocol was implemented in patient group A, or the target group (heart rate (HR), bpm; blood pressure (BP), mmHg; peripheral oxygen saturation, SpO_2_, %; capnography, EtCO_2_, mmHg; state entropy (SE); response entropy (RE); inspired oxygen fraction, FiO_2_; minimum alveolar concentration (MAC)); in group B, or the control group, general anesthesia was guided based on standard procedure (inspired oxygen fraction, FiO_2_). Based on the study protocol, the patient inclusion criteria were as follows: age—over 18; gender—male and female; surgical procedure—laparoscopic cholecystectomy; inhalational general anesthesia with Sevoflurane. The exclusion criteria were pregnancy, septic shock, massive hemorrhage, ketamine administration, and total intravenous anesthesia (TIVA). Patient allocation to the study group was randomized using online software (http://www.randomization.com).

### 2.2. Measurements and Data Management

Study data were processed from the prior approved monitoring form. The data were filed electronically by the “data officer”, later being de-identified and secured by password in the study database. The study database included demographical and clinical data of all patients in the study, as well as values of the monitored parameters based on protocol, as follows: individual patient code, gender, age, ASA score, type of surgery, surgery duration, RE and SE values, heart rate, systolic blood pressure, peripheral oxygen concentration, minimum alveolar concentration, gas flow, inspired oxygen fraction, and number of hemodynamic events (hypertension, hypotension, tachycardia, bradycardia). Doses of used anesthetic drugs were also recorded for Fentanyl, Propofol, Rocuronium, Sevoflurane, vasopressor drugs, and maintenance fluids given in the perioperative period. Regarding the hemodynamic parameters, data recording was carried out based on the following scheme: starting time before orotracheal intubation (M0), followed by continuous recordings every 15 min (M15, M30, […]), with the final recording at ± 5 mins before extubation (Mext).

For the characteristic hemodynamic events, they were considered as follows: hypotension if systolic blood pressure dropped under 70 mmHg; hypertension if systolic blood pressure increased by over 20% compared to the start value; bradycardia for heart rate under 45 bpm; and tachycardia for heart rate over 100 bpm. For study group A, general anesthesia (which was maintained in the 40–60 interval, based on current guidelines) was optimized based on the entropy values. In study Group B, general anesthesia was guided based on classical schemes of drug dosing and optimization. 

### 2.3. General Anesthesia and Monitoring

After admission to the operating room (OR), all patients were monitored using a standard monitor (Carescape B650, GE Healthcare, Helsinki, Finland). SE and RE were monitored using the same device with the entropy module attached (E-Entropy Module, GE Healthcare, Helsinki, Finland). Entropy sensors were placed on the forehead of patients in Group A, based on the producer’s guidelines. During induction, all patients received the same drugs based on local protocols. Mechanical ventilation and hypnosis were achieved through continuous administration of Sevoflurane, using the same anesthesia machine (Avance CS2, GE Healthcare, Chicago, IL, USA) for all patients (i.e., in both groups A and B).

### 2.4. Statistical Analysis

All clinical data were registered in the electronic study database by the “data officer”. The GraphPad 7 software (Graphpad Software Incorporated, San Diego, CA, USA) was used for the statistical analysis. Regarding the statistical methodology for quantitative values, we calculated the mean and standard deviation; while, for non-quantitative values, we calculated frequency and percentage. The 95% confidence interval (95% CI) was also presented as an argument for statistical differences. The Student’s t-test (normal distribution) and the Mann–Whitney U test (non-normal distribution) were applied for comparison between numerical values. Multiple comparisons were carried out using the one-way ANOVA test. Statistically significant differences were considered for p < 0.05. 

## 3. Results

### 3.1. Clinical and Demographical Characteristics

**Figure 1:**
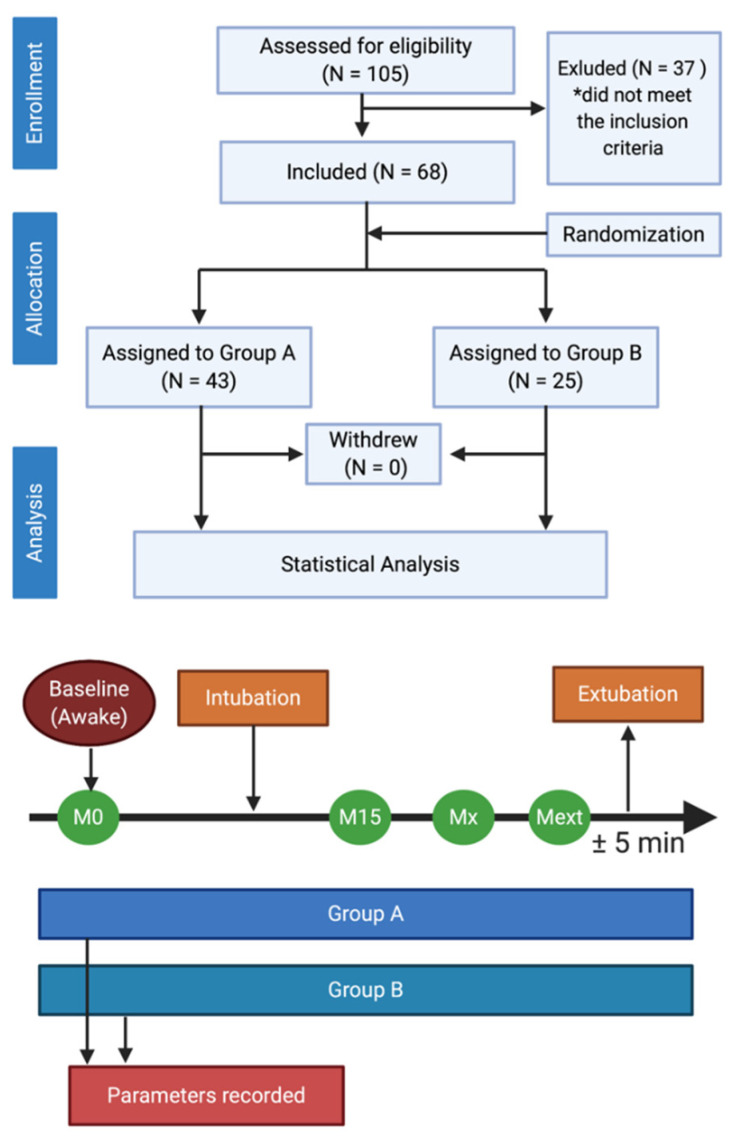
Study flowchart and data processing methodology.

Between January and December 2019, 68 patients were identified as eligible for the study, based on the inclusion and exclusion criteria. The total number of patients presenting with the studied pathology was N = 105; however, a number of these were excluded from the study protocol. After applying the randomization protocol, 43 patients were allocated to group A and 25 patients to group B. No particular events were recorded in either of these groups that could have led to the exclusion of certain patients from the study (Figure 1).

For further statistical evaluation, we first compared demographic and clinical data (Table 1) of patients in groups A and B, and found no statistically significant differences. The Chi-square test with 1 degrees of freedom (d.f.) was used to analyze the gender distribution. The Student’s t-test (two-tailed, unpaired) was used for comparison of the other values. A confidence interval (95%) is also presented for all analyzed characteristics. 

### 3.2. State Entropy and Response Entropy Expression

**Figure 2:**
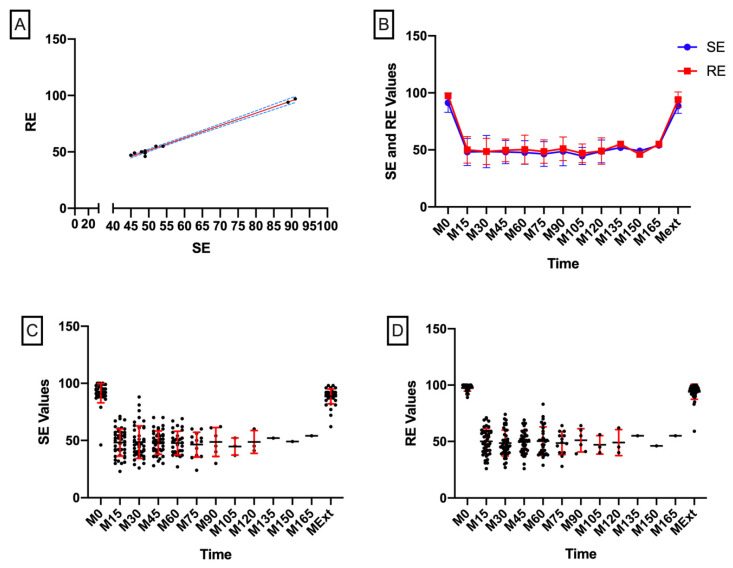
State Entropy (SE) and response entropy (RE) expression. (**A**) correlation between SE and RE values (p = 0.7620, 95% CI: [0.9812, 0.9982], r = 0.9942, R^2^ = 0.9884); (**B**) evolution of SE and RE in time; (**C**) expression of SE during general anesthesia (black dots, individual subject measurements; red lines, standard deviation; black lines, means); (**D**) expression and allocation of SE in time during general anesthesia (black dots, individual subject measurements; red lines, standard deviation; black lines, means).

After induction of general anesthesia in Group A, we observed a decrease in the value of both SE and RE. At M0, the mean values for SE and RE were 91.37 vs. 97.47 (mean difference: −6.100). After orotracheal intubation and stabilization of the degree of hypnosis, the mean values for SE and RE came progressively closer to being equal (M15: 48.14 vs. 50.07, mean difference: −1.930; M30: 48.56 vs. 48.60, mean difference: −0.0400). Interestingly, after reducing the Sevoflurane concentration and the degree of hypnosis, the difference between the mean SE and RE increased to the initial value. Following this trend, before extubation (i.e., at Mext), the mean values for SE and RE reached 88.60 vs. 94.09 (mean difference: −5.490), as shown in Figure 2.

**Figure 3:**
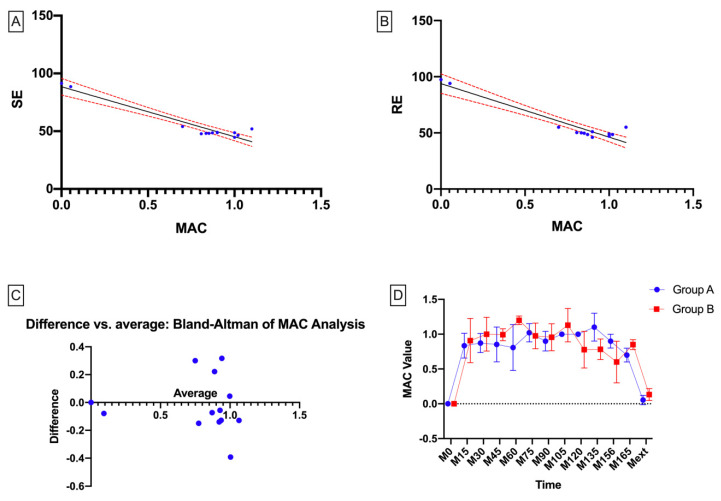
Statistical correlations for minimum alveolar concentration (MAC) and SE/RE. (**A**) correlations for SE and MAC (r = −0.9583, r^2^ = 0.9184, 95% CI: [−0.9878, −0.8630]); (**B**) correlations for RE and MAC (r = −0.9519, r^2^ = 0.9043, 95% CI: [−0.9855, −0.8402]); (**C**) Bland–Altman analysis of MAC values; and (**D**) MAC values over time.

An important aspect of the monitoring of general anesthesia is the minimum alveolar concentration (MAC) of a volatile anesthetic agent. Interestingly enough, in our study, the mean MAC value after the first 15 mins of general anesthesia was 0.8349 for Group A vs. 0.9080 for Group B. Based on the statistical analysis between MAC values and SE (respectively, MAC values and RE) in Group A, there was no statistical correlation (Figure 3). There was a statistically significant difference for the correlation between MAC values in the two groups (Group A vs. Group B; p = 0.0008, r = 0.8112, R^2^ = 0.6580, 95% CI: [0.4705, 0.9414]).

**Figure 4:**
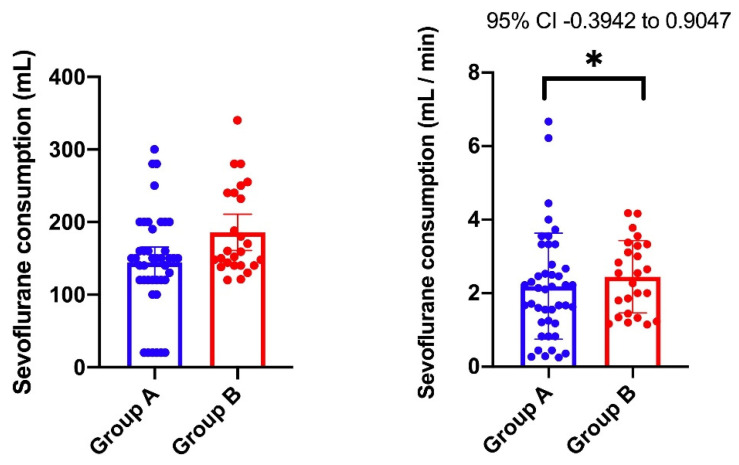
Statistical analysis for sevoflurane consumption. (**Left**) scatter plot with bar (mean with SD) for total consumption of Sevoflurane (mL), (group A: lower 95% CI of mean 122.5, upper 95% CI of mean 165.6, coefficient of variation 47.90%; group B: lower 95% CI of mean 160.9, upper 95% CI of mean 210.7, coefficient of variation 32.47%); (**Right**) scatter plot with bar (mean with SD) for Sevoflurane consumption/minute (mL/min), (group A: lower 95% CI of mean: 1.748, upper 95% CI of mean: 2.634, variation coefficient: 65.73%; group B: lower 95% CI: 2.040, upper 95% CI: 2.853, variation coefficient: 40.26%). The mean difference between the two groups was 0.2553 ± 0.3253 and the 95% CI was [−0.3942, 0.9047].

Regarding Sevoflurane consumption, Group A showed a mean consumption of 144.0 ± 69.00 mL compared to Group B, in which the mean was 185.8 ± 60.33 mL. Volatile anesthetic consumption reported to time expressed statistically significant differences between the two groups. Group A had a mean consumption of 2.191 ± 1.440 mL/min (lower 95% CI of mean: 1.748, upper 95% CI of mean: 2.634, variation coefficient: 65.73%) vs. 2.446 ± 0.9849 mL/min in Group B (lower 95% CI: 2.040, upper 95% CI: 2.853, variation coefficient: 40.26%). The mean difference between the two groups was 0.2553 ± 0.3253 and the 95% CI was [−0.3942, 0.9047]. Statistically significant differences were identified between the two groups regarding the consumption of anesthetic gas, with group A having a lower threshold (p = 0.0498), as shown in Figure 4.

### 3.3. Hemodynamic Stability During Surgery

Hemodynamic stability was assessed based on a number of different parameters. These parameters included heart rate (HR, bpm), systolic blood pressure (mmHg), and the number of recorded hemodynamic events (e.g., hypotension, hypertension, bradycardia, and tachycardia). In Group A, hemodynamic events were recorded in a number of 1.6/N (N = 43), of which 17 (24.4%) were hypertension, 19 (28.4%) were hypotension, 12 (17.9%) were tachycardia, and 19 (28.4%) were bradycardia. In Group B 2.84/N (N = 25) hemodynamic events were recorded, of which 21 (29.6%) were hypertension, 14 (19.7%) were hypotension, 21 (29.6%) were tachycardia, and 15 (21.1%) were bradycardia. For a more complete record of the number of adverse hemodynamic events, these are reported, in terms of the number of patients in each group, in Table 2.

**Figure 5:**
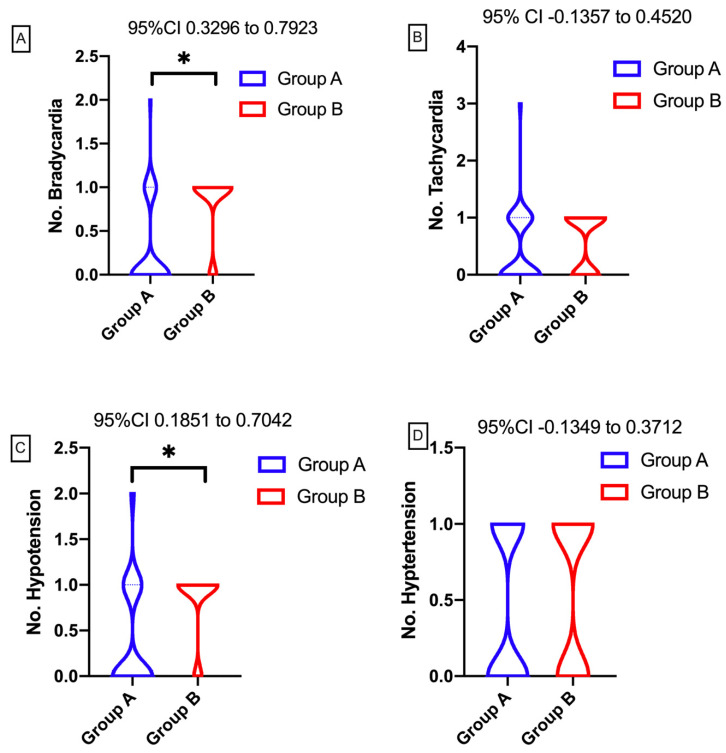
Statistical and graphical analyses of perioperative hemodynamic changes. (**A**) number of bradycardia episodes; (**B**) number of tachycardia episodes; (**C**) number of hypotensive episodes; and (**D**) number of hypertensive episodes. Regarding the statistical analysis of the intraoperative hemodynamic events, significant statistical differences can be observed in the number of bradycardia (A) events (respectively, in the number of hypotension (C) events), where there was a decrease in the incidence for patients in group A. In contrast, regarding the number of tachycardia (B) events (respectively, of the number of hypertension (D) events), no statistically significant differences were observed between the two groups.

For the statistical analysis of the two groups, the results show a significantly lower number of hypotensive events in group A (p = 0.011; 95% CI: [0.1851, 0.7042]; min 0, max 2; 25% percentile: 0, 75% percentile: 1; range: 2). Statistically significant differences were noticed for bradycardia, with a decreased incidence in group A (p < 0.0001; 95% CI: [0.3296, 0.7923]; min 0, max 1; 25% percentile: 1, 75% percentile: 1; range: 1). There were no statistically significant differences for hypertensive events (p = 0.3547; 95% CI: [−0.1349, 0.3712]; min: 0, max: 1; 25% percentile: 0, 75% percentile: 1; range: 1) or for tachycardia (p = 9.2866; 95% CI: [−0.1357, 0.4520]; min: 0, max: 1; 25% percentile: 0, 75% percentile: 1; range: 1), as shown in Figure 5. The distribution for the number of events in each group shows that, in group A, most patients experienced no bradycardia, a very low number of patients experienced 1 episode (N = 10, 83.33%), and two bradycardia events were recorded only in an isolated case (N = 1, 8.37%). On the other hand, in group B, an increased number of patients experienced at least one episode of bradycardia (N = 21, 84%). The hemodynamic changes represented by hypotension followed a similar trend. Most of the patients in Group A (76.47%) presented only one blood pressure drop (hypotension), while 11.77% presented two hypotensive episodes. In contrast, 86% of patients in group B presented one episode of hypotension. Although from a distribution perspective, tachycardia and hypertension events were different, these differences were not statistically significant (Figure 5).

**Figure 6:**
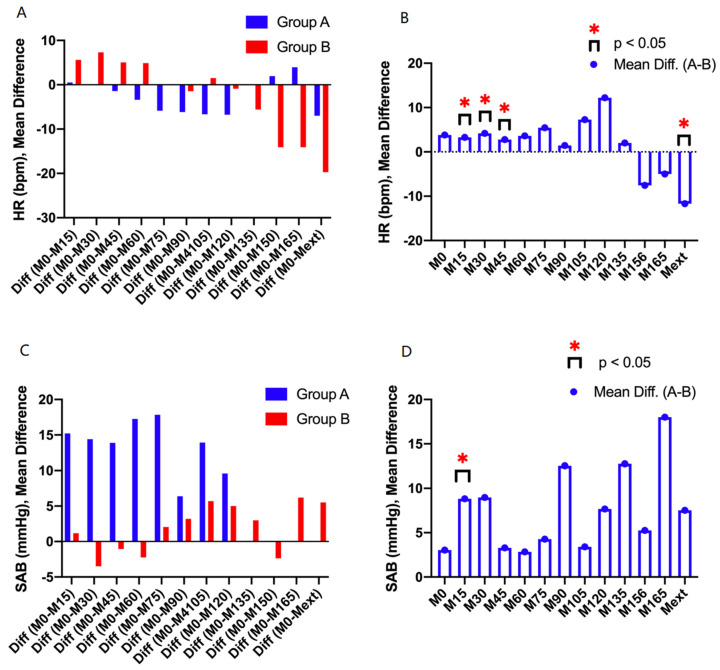
(**A, B**) Statistical analysis of mean differences for heart rate (HR, bpm); (**C, D**) Statistical analysis of mean differences for blood pressure (SAB, mmHg).

Analysis of the heart rate (HR, bpm) dynamics in the two groups revealed important statistical variations. In Group A, there were statistically significant differences recorded for the hemodynamic changes between M0 and M15 (p < 0.05), M0 and M30 (p < 0.05), and M0 and Mext (p < 0.05). A similar tendency was recorded for group B, with significant differences between M0 and M15 (p < 0.05), M0 and M30 (p < 0.05), and M0 and Mext (p < 0.05). For systolic blood pressure (SAB, mmHg), differences were statistically significant in group A between M0 and M15 (p < 0.05), M30 (p < 0.05), M45 (p < 0.05), and M60 (p < 0.05). In group B, significant differences were recorded for a longer time span, as follows: M0 and M15 (p < 0.05), M30 (p < 0.05), M45 (p < 0.05), M60 (p < 0.05), M75 (p < 0.05), M90 (p < 0.05), M105 (p < 0.05), M120 (p < 0.05), M135 (p < 0.05), and M150 (p < 0.05) (see Table 3 and Figure 6).

Regarding the awakening and recovery time for an Aldrete score over 9, no statistically significant differences were identified between groups (p < 0.05).

## 4. Discussion

Common technologies for monitoring the degree of hypnosis/depth of anesthesia are represented by BIS, entropy (SE/RE), and the Narcotrend index. From a technical point of view, BIS is based on the analysis of EEG variations. Bispectral analysis is characterized by the sub-variable called “SynchFastShow”, which is mathematically defined as the logarithm of the sum of all bispectral peaks in the 0.5–47 Hz interval. The Narcotrend index is based on EEG classification at different stages, based on the degree of hypnosis. In this manner, the stages are classified from A (awake) to F (very deep level of anesthesia). The technology is based on the statistical analysis of EEG signals associated with the stages that indirectly account for the depth of anesthesia. In our study, the patients in Group A that received multimodal monitoring had a mean value for SE and RE in the reference interval of 40–60 [6,7,8,9]. In clinical practice, there are numerous cases where modulating the depth of anesthesia becomes imperative, especially in the case of elderly patients with many comorbidities. Such a group is represented by patients proposed for cardiac surgery, where higher medication doses lead to increased hemodynamic instability. This field has drawn attention to patients needing cardiopulmonary bypass, as these patients are usually also receiving beta-blockers and other hypotensive medication. In these situations, classical monitoring parameters are no longer reliable for monitoring the depth of anesthesia [10]. Another important fact is that recent studies have shown that there is not a strong correlation between BIS and entropy monitoring in these situations. Lehmann et al. have reported a lack of correlation between BIS and entropy [11]. A similar study has been carried out by Meybohm et al., showing a very important aspect for cardiac surgery with cardiopulmonary bypass regarding hypothermia. The group has reported significant correlations between BIS and entropy under the conditions of normothermia (BIS vs. SE: r^2^ = 0.56; BIS vs. RE: r^2^ = 0.58), but lower correlations between the two parameters for hypothermic patients (BIS vs. SE: r^2^ = 0.17; BIS vs. SE: r^2^ = 0.18) [12]. Musialowicz et al. have also shown low correlations between BIS and entropy in patients under cardiopulmonary bypass [10]. Ma et al. carried out a study regarding the impact of anesthetic drug doses in cardiac surgery with cardiopulmonary bypass. They reported a significant decrease in the propofol and sufentanyl consumption in the case of patients who received entropy monitoring (p < 0.05). Another important factor that Ma et al. have reported is the positive clinical impact on hemodynamic stability in the case of entropy monitoring, with their control group needing higher vasopressor doses (p < 0.05) [13].

From recent clinical practice, it is a known fact that surgical procedures involving a laparotomy imply a longer and more difficult recovery. These patients more often experience a series of adverse phenomena, such as lack of energy, prolonged fatigue, and a more difficult reintegration into their day-to-day lives. Apart from these, the literature has reported other side effects after surgery, such as pain, longer periods of analgesic medication use, nausea and vomiting, loss of appetite, increased bleeding risk, and increased risk for infection. These are some of the reasons why surgical techniques have evolved in the last decade, with laparoscopic surgery becoming routine practice worldwide. Once this new technique was used at a larger scale, a series of aspects regarding general anesthesia arose as important parameters for monitoring. One important aspect associated with general anesthesia for laparoscopic surgery is that of perioperative respiratory and ventilatory dysfunctions, such as volutrauma, barotrauma, and atelectasis. The main cause of these complications is increased intra-abdominal pressure, which pushes the diaphragm and doubles the pressure in the thoracic cavity. By maintaining a higher-than-normal intra-abdominal pressure, the main factors which favor complications are increased pressure, the position of the patient, and carbon dioxide absorption [14].

In our study, one of the main objectives was to monitor the impact of general anesthesia on hemodynamic stability during this type of procedure. The hypothesis was that modulating the anesthetic doses based on the individual needs of each patient might have a positive impact on perioperative hemodynamic stability. In other words, our study focused on optimizing the Sevoflurane dose by monitoring the degree of hypnosis/depth of anesthesia based on the entropy (i.e., SE and RE). The main mechanisms influencing the hemodynamic stability of patients that undergo laparoscopic surgery are the impacts on venous return and myocardial contractility, and the increase in systemic vascular resistance [15]. A high percentage of these patients have decreased cardiac output with hemodynamic collapse due to increased intra-abdominal pressure, which leads to the compression of the inferior vena cava and further decrease of venous return. Furthermore, the increased vascular resistance leads to an increased motor load of the heart with tachycardia which can lead to hemodynamic collapse [16,17,18,19,20]. It is well-known that one of the pharmacodynamic effects of volatile anesthetics is their impact on the heart function [21,22,23], but it should be noted that, apart from its effects on the hemodynamic status, Sevoflurane also has cardioprotective properties. From a molecular point of view, the cardioprotective effects are attained through its action on adenosine triphosphate-sensitive channels which can be found in the cardiac myocytes. Tanaka et al. have shown that Isoflurane can have a beneficial cardiac pre-conditioning effect by modulating mitochondrial potassium channels after the activation of adenosine triphosphate [24]. Beneficial effects on the clinical prognosis have also been shown for Propofol. Recent studies have shown that total intravenous general anesthesia (TIVA) with Propofol is associated with fewer post-operative side-effects [25,26,27]. Among these are a decrease in post-operative pain, modulation of the cerebral blood flow, a decrease in intracranial pressure, and a lower incidence of post-operative nausea and vomiting. The study carried out by Kawano et al., on the implications of Propofol and Sevoflurane on the post-operative side-effects concluded that, by using this drug combination, the incidence of specific side-effects is significantly lower [28]. In our study, we have shown that, by optimizing the doses of inhalational anesthetic guided by entropy, we can obtain better hemodynamic stability. In the patients included in the multimodal study group (Group A), we noticed a statistically significant decrease in the incidence of hypotensive episodes (p < 0.05), as well as in the incidence of bradycardia (p < 0.05). No implications were noticed for tachycardic or hypertensive episodes (p > 0.05).

A series of recent studies and recommendations have proven the need for implementing multimodal monitoring protocols in general anesthesia for laparoscopic surgery. Most of them have referred to hemodynamic and respiratory monitoring. In recent years, there have been extensive debates on introducing, as routine, monitoring protocols for parameters capable of determining the depth of anesthesia, the nociception–antinociception balance, and neuromuscular transmission [29,30,31]. In regard to hemodynamic monitoring, the balance favors the invasive monitoring of arterial blood pressure. In the case of patients with associated cardiovascular comorbidities, monitoring techniques are more complex, bringing vital information on the filling pressure of the heart, the preload, and systemic pressure distribution. Regarding mechanical ventilation, in the case of patients undergoing laparoscopic surgery, pressure-based ventilation is preferred. Moreover, PEEP titration based on the clinical context reduces the specific side-effects of alveolar collapse [32]. More attention should be given to the cardiovascular stability of these patients and to the continuous adaptation of the PEEP value based on their hemodynamic profile [33,34,35].

Numerous studies have shown a series of correlations between BIS and the anesthetic dose, the incidence of side-effects, and recovery time, but only a few have shown a strong statistical correlation between the plasmatic concentration of anesthetic drugs and the BIS signal expression [8,36]. This fact serves only to confirm the need for an individualized approach towards general anesthesia, based on the particular needs of each patient. Shah et al. carried out a study on the impact that entropy monitoring can have on the hemodynamic status and reported a positive influence on hemodynamic stability, as well as a reduction in anesthetic doses [37]. A similar study carried out by Riad et al. reported a reduction in Propofol doses by 37.1% in the entropy monitoring group, compared to the control (p < 0.05) [38]. Tewari et al. studied the recovery time and anesthetic drug doses in patients which underwent transvaginal oocyte retrieval under general anesthesia. The results of the study group proved that entropy monitoring led to a decreased Propofol consumption, by 6.7% (p = 0.01). They also reported lower opioid (Fentanyl) doses (p = 0.007). An important aspect has been noted in the recovery room where, in the case of the group with entropy monitoring, only 10% of patients needed supplemental post-operative analgesia, compared to the control group, where 28.3% of patients needed supplementation (p = 0.01) [39]. Wu et al. also reported a statistically significant decrease (p ≤ 0.05) for Sevoflurane consumption in the entropy group (27.79 ± 7.4 mL vs. 31.42 ± 6.9 mL). Furthermore, they proved its positive impact on hemodynamic stability compared to the control group (p = 0.043) [40]. In our study, we have identified a similar trend, as Group A had significantly lower Sevoflurane consumption compared to the control group (p = 0.0498). Another study on the impact of entropy on anesthetic drug doses was carried out by Vakkuri et al., reporting significant differences between their study groups with a positive impact on recovery time in the case of patients benefitting from entropy monitoring [41]. El Hor et al. carried out a similar study on patients undergoing laparoscopic rectosigmoidectomy. They reported decreased Sevoflurane consumption in the group where general anesthesia was optimized by using the entropy (5.2 ± 1.4 mL/h vs. 3.8 ± 1.5 mL/h; p = 0.0012). In the same group, they reported improved hemodynamic stability and a decrease in hypotension incidence (p = 0.03) [42].

Post-operative recovery is another very important aspect, both from the functional and the cognitive points of view [43]. It is a well-known fact that surgical stress and general anesthesia can impact cognitive and neurological recovery. Zhang et al. carried out a study on the impact of Sevoflurane and Propofol on neuro-cognitive recovery. They concluded that intravenous anesthesia with Propofol has fewer side-effects than inhalational anesthesia with Sevoflurane [44]. This is another essential argument for introducing hypnosis monitoring based on EEG analysis. Kadoi et al. demonstrated a minimization of neurological impact in the case of intravenous anesthesia with Propofol in the elderly [45]. A similar study carried out by Radtke et al. found similar results on the implications of depth of anesthesia monitoring on post-operative recovery. They observed a delirium incidence of only 16.7% in the group where the depth of anesthesia was monitored, compared with 21.5% in the control group (p = 0.036). They also showed the lack of cognitive imbalance later on, but with no significant differences at 7 days (p = 0.062) and at 90 days (p = 0.372) [46]. Post-operative complication rate has been shown to vary between 20% and 42% [47]; they have an impact both on the clinical outcome of patients as well as on the OR management and patient outflow [48]. This is due to longer times of stay in the recovery room and a longer hospital length of stay [49,50,51,52]. Another important aspect that is directly connected to post-operative complications is the economical aspect of the medical system, with increased costs in such situations. The main cause of secondary complications is hemodynamic instability, with direct effects on dopaminergic, muscarinic, and serotoninergic receptors. The central and peripheral opioid receptors are also affected by both over- or under-dosing of anesthetic drugs [53,54,55,56,57].

One limitation of these monitoring techniques is the lack of evidence in pediatric patients. Both entropy and BIS have presented low statistical correlations in the pediatric population under 1 year of age. Davidson et al. studied the performance of BIS and entropy in different pediatric groups. They have designed different study groups for certain age intervals: 0–1 years, 1–2 years, 2–4 years, and 4–12 years. Following their study, they concluded that, for children under the age of 1, there are large differences between the measurements both for entropy and for BIS. They did not report significant differences between the two technologies [58]. Another limitation of both technologies is with the concomitant administration of ketamine [59]. Once ketamine is administered, the value of both parameters are increased and the correlations are no longer valid. Hans et al. have studied the effects induced by ketamine on the depth of anesthesia monitoring techniques and have reported a significant change in the expression of BIS, SE, and RE [60].

Another very important consideration is represented by the environmental impact of volatile anesthetics. From a chemical point of view, volatile anesthetic gases are organic halogenated compounds that negatively impact the ozone layer. Moreover, they persist in the atmosphere for long periods of time, contributing to global warming. Brown et al., in a study on the impact of the anesthetic gases halotane, desflurane, and enflurane on the ozone layer, concluded that their remanence times are 2.5 to 21.4 years [61]. The volumes used seem small, but pharmaceutical companies deliver millions of liters of volatile anesthetics worldwide annually. A recent study estimated that a medium-sized hospital in the U.S. uses around 1000 L of volatile anesthetic gases yearly [62].

The limitations of our study are the lack of monitoring for the nociception–antinociception balance and that no correlations have been made between this parameter and the consumption of Fentanyl. Last, but not least, the study did not focus on post-operative recovery times of more than 4 h after post-anesthesia care unit (PACU) and no data have been collected on the neurocognitive recovery at 24 and 48 h.

## 5. Conclusions

In conclusion, we can say that multimodal monitoring which includes both classical parameters and monitoring of the depth of anesthesia through entropy improved perioperative hemodynamic stability. Our study demonstrated a decreased incidence of both hypotension and bradycardia episodes in the group which benefited from multimodal monitoring with personalized anesthetic dosage based on the entropy value. Furthermore, we recorded a decrease in Sevoflurane consumption in the group where general anesthesia was optimized by entropy.

We can conclude that, by adapting the general anesthetic technique based on the individual needs of each patient, clinicians can achieve individualized anesthesia with a significant positive impact on perioperative hemodynamic stability and on the consumption of volatile anesthetics. Finally, we can underline the increase in patient safety and improved therapeutic management by adapting current practices towards personalized medicine, tailored to the individual needs of each patient.

## Figures and Tables

**Table 1:** Clinical and demographical characteristics of the study groups.

Characteristic	Group A (N = 43)	Group B (N = 25)	95% Confidence Interval	Statistical p Value
Age; years, mean ± SD	51 ± 16.51	52.20 ± 13.79	−6.620–9.020	> 0.05
Weight; kg, mean ± SD	87 ± 2.71	91 ± 1.99	−5.445–7.012	> 0.05
Gender; M, N (%)	7 (16.28)	6 (24)	−10.8233%–28.7947%	> 0.05
ASA Score; I, N (%)	10 (23)	3 (12)	−0.5716%–27.4520%	> 0.05
ASA Score; II, N (%)	24 (56)	17 (68)	−11.9231%–32.8672%	> 0.05
ASA Score; III, N (%)	6 (14)	5 (20)	−11.3628%–26.5172%	> 0.05
HR at M0; bpm, mean ± SD	78.48 ± 13.87	75.32 ± 14.28	−10.46–3.616	> 0.05
SAB at M0; bpm, mean ± SD	136.5 ± 22.47	134 ± 17.51	−12.97–7.917	> 0.05

SD, standard deviation; M, male; N, number of patients; HR, heart rate; SAB, systolic blood pressure; M0, time before intubation/moment 0; p, statistically significant for p < 0.05.

**Table 2:** Hemodynamic changes in group A and group B.

	Group A (N = 43)			Group B (N = 25)		
	No. Hemodynamic Events	No. Hemodynamic Events/Patient	% of Hemodynamic Events	No. Hemodynamic Events	No. Hemodynamic Events/Patient	% of Hemodynamic Events
No. Hypertensions	17	0.4	25.4	21	0.84	29.6
No. Hypotensions	19	0.5	28.4	14	0.56	19.7
No. Tachycardia	12	0.3	17.9	21	0.84	29.6
No. Bradycardia	19	0.5	28.4	15	0.6	21.1
Total	67	1.6		71	2.84	

**Table 3:** Statistical analysis of the dynamics for heart rate and blood pressure.

HR (bpm)														
		M0	M15	M30	M45	M60	M75	M90	M105	M120	M135	M156	M165	Mext
Group A	MEAN	75.9	75.4	76.1	77.3	79.3	81.8	82.1	82.6	82.7	76.0	74.0	72.0	82.9
	SD	13.-9	11.7	12.1	11.3	13.4	12.5	12.2	16.8	10.8	0.0	0.0	0.0	9.8
	p		M15/M0	M30/M0	M45/M0	M60/M0	M75/M0	M90/M0	M105/M0	M120/M0	M135/M0	M150/M0	M165/M0	Mext/M0
			< 0.05	< 0.05	> 0.05	< 0.05	> 0.05	> 0.05	> 0.05	> 0.05	-	-	-	< 0.05
Group B	MEAN	74.9	69.3	67.6	69.9	70.0	75.0	76.4	73.4	75.8	80.5	89.0	89.0	94.6
	SD	14.4	6.3	7.7	9.6	8.9	9.1	8.3	8.8	9.5	10.9	11.1	9.9	18.2
	p		M15/M0	M30/M0	M45/M0	M60/M0	M75/M0	M90/M0	M105/M0	M120/M0	M135/M0	M150/M0	M165/M0	Mext/M0
			< 0.05	< 0.05	> 0.05	> 0.05	> 0.05	> 0.05	> 0.05	> 0.05	> 0.05	< 0.05	> 0.05	< 0.05
SAP (mmHg)														
Group A	MEAN	136.6	121.4	122.2	122.7	119.3	118.8	130.2	122.7	127.0	0.0	0.0	0.0	136.6
	SD	23.9	24.1	19.8	17.9	14.6	15.1	23.8	4.6	5.2	0.0	0.0	0.0	15.7
	p		M15/M0	M30/M0	M45/M0	M60/M0	M75/M0	M90/M0	M105/M0	M120/M0	M135/M0	M150/M0	M165/M0	Mext/M0
			< 0.05	< 0.05	< 0.05	0< 0.05	< 0.05	> 0.05	> 0.05	> 0.05	-	-	-	> 0.05
Group B	MEAN	134.0	109.9	112.0	118.5	117.7	113.9	112.6	116.6	115.0	114.3	117.8	105.0	129.4
	SD	17.5	16.3	21.0	18.6	19.7	15.5	14.3	11.8	12.5	14.5	19.9	11.3	12.0
	p		M15/M0	M30/M0	M45/M0	M60/M0	M75/M0	M90/M0	M105/M0	M120/M0	M135/M0	M150/M0	M165/M0	Mext/M0
			< 0.05	< 0.05	< 0.05	< 0.05	< 0.05	< 0.05	< 0.05	< 0.05	< 0.05	< 0.05	> 0.05	> 0.05

## References

[1] Kanakala V., Borowski D.W., Pellen M.G.C., Dronamraju S.S., Woodcock S.A.A., Seymour K., Attwood S.E.A., Horgan L.F. (2011). Risk factors in laparoscopic cholecystectomy: A multivariate analysis. Int. J. Surg..

[2] Ellakany M. (2013). Comparative study between general and thoracic spinal anesthesia for laparoscopic cholecystectomy. Egypt. J. Anaesth..

[3] Wycherley A.S., Bembridge J.L. (2017). Monitoring techniques; neuromuscular blockade and depth of anaesthesia. Anaesth. Intensive Care Med..

[4] Ozdogan H.K., Cetinkunar S., Karateke F., Cetinalp S., Celik M., Ozyazici S. (2016). The effects of sevoflurane and desflurane on the hemodynamics and respiratory functions in laparoscopic sleeve gastrectomy. J. Clin. Anesth..

[5] Rogobete A.F., Bedreag O.H., Sandesc D. (2017). Entropy-Guided Depth of Anesthesia in Critically Ill Polytrauma Patients. J. Interdiscip. Med..

[6] Cotae A., Grinţescu I.M. (2019). Entropy—The Need of an Ally for Depth of Anesthesia Monitoring in Emergency Surgery. Cent. Eur. Ann. Clin. Res..

[7] Shalbaf R., Behnam H., Sleigh J.W., Steyn-Ross A., Voss L.J. (2013). Monitoring the depth of anesthesia using entropy features and an artificial neural network. J. Neurosci. Methods.

[8] Bruhn J., Myles P.S., Sneyd R., Struys M.M.R.F. (2006). Depth of anaesthesia monitoring: What’s available, what’s validated and what’s next?. Br. J. Anaesth..

[9] Ortolani O., Conti A., Di Filippo A., Adembri C., Moraldi E., Evangelisti A., Maggini M., Roberts S.J. (2002). EEG signal processing in anaesthesia. Use of a neural network technique for monitoring depth of anaesthesia. Br. J. Anaesth..

[10] Musialowicz T., Lahtinen P., Pitkänen O., Kurola J., Parviainen I. (2011). Comparison of Spectral Entropy and BIS VISTA^TM^ monitor during general anesthesia for cardiac surgery. J. Clin. Monit. Comput..

[11] Lehmann A., Schmidt M., Zeitler C., Kiessling A.-H., Isgro F., Boldt J. (2007). Bispectral index and electroencephalographic entropy in patients undergoing aortocoronary bypass grafting. Eur. J. Anaesthesiol..

[12] Meybohm P., Gruenewald M., Höcker J., Renner J., Graesner J.-T., Ilies C., Scholz J., Bein B. (2010). Correlation and agreement between the bispectral index vs. state entropy during hypothermic cardio-pulmonary bypass. Acta Anaesthesiol. Scand..

[13] Ma J., Wang X., Xie Y., Yu J., He Q., Li Z., Du J., Jiang X. (2012). Spectral entropy monitoring reduces anesthetic dosage for patients undergoing off-pump coronary artery bypass graft surgery. J. Cardiothorac. Vasc. Anesth..

[14] Vargas M., Brunetti I., Pelosi P. (2013). Protective mechanical ventilation during general anaesthesia. Trends Anaesth. Crit. Care.

[15] Kim D., Ahn J.H., Jung H., Choi K.Y., Jeong J.S. (2019). Effects of neuromuscular blockade reversal on bispectral index and frontal electromyogram during steady-state desflurane anesthesia: A randomized trial. Sci. Rep..

[16] De Rosa R.C., Romanelli A., Calabria M., Abbate R., Montesano R., Corcione A. (2019). Continuous Noninvasive Haemoglobin Monitoring in Vascular Surgery within the Goal-Directed Therapy Protocol. CEACR.

[17] Cotrau P., Hodosan V., Vladu A., Timar C., Daina L., Pantis C., Negrau M., Daina C., Vernic C. (2019). Occupational Stress and Burnout Syndrome among ICU Nurses. A Prospective Observational Study. CEACR.

[18] Moise A., Balescu-arion C. (2020). The Vitamin D and the Immune System. When? Why? How?. Cent. Eur. Ann. Clin. Res..

[19] Georgescu D., Reisz D., Petre I., Ionita I. (2019). Ischemic Stroke Secondary to Cerebral Venous Thrombosis: A Case Report. Cent. Eur. Ann. Clin. Res..

[20] Colombo R., Raimondi F., Rech R., Castelli A., Fossali T., Marchi A., Borghi B., Corona A., Guzzetti S. (2015). Surgical Pleth Index guided analgesia blunts the intraoperative sympathetic response to laparoscopic cholecystectomy. Minerva Anestesiol..

[21] Xie P., Li Z., Tian Z. (2016). The optimal combination of mechanical ventilator parameters under general anesthesia in obese patients undergoing laparoscopic surgery. J. Clin. Anesth..

[22] Feldman L.S., Kaneva P., Demyttenaere S., Carli F., Fried G.M., Mayo N.E. (2009). Validation of a physical activity questionnaire (CHAMPS) as an indicator of postoperative recovery after laparoscopic cholecystectomy. Surgery.

[23] Papurica M., Sandesc D., Rogobete A.F., Nartita R., Vernic C., Popovici S.E., Bedreag O.H. (2016). Cardioprotective Effects Induced by Preconditioning with Halogenated Anesthetics. J. Interdiscip. Med..

[24] Tanaka K., Weihrauch D., Ludwig L.M., Kersten J.R., Pagel P.S., Warltier D.C. (2003). Mitochondrial adenosine triphosphate-regulated potassium channel opening acts as a trigger for isoflurane-induced preconditioning by generating reactive oxygen species. Anesthesiology.

[25] Metry A.A., Hussain N.S., Nakhla G.M., Ragaei M.Z., Wahba R.M. (2019). The effect of continuous propofol versus dexmedetomidine infusion on regional cerebral tissue oxygen saturation during cardiopulmonary bypass. Rom. J. Anaesth. Intensive Care.

[26] Riazanova O.V., Alexandrovich Y.S., Guseva Y.V., Ioscovich A.M. (2019). A randomized comparison of low dose ropivacaine programmed intermittent epidural bolus with continuous epidural infusion for labour analgesia. Rom. J. Anaesth. Intensive Care.

[27] Balan S.A., Bubenek-turconi Ş.I., Droc G., Marinescu E., Nita E., Popa C., Popescu-spineni D., Tomescu D. (2019). Burnout syndrome in the Anaesthesia and Intensive Care Unit. Rom. J. Anaesth. Intensive Care.

[28] Kawano M., Tanaka K., Itonaga I., Iwasaki T., Tsumura H. (2015). C-Myc represses tumor-suppressive microRNAs, let-7α, miR-16 and miR-29b, and induces cyclin D2-mediated cell proliferation in Ewing’s sarcoma cell line. PLoS ONE.

[29] Maestroni U., Sortini D., Devito C., Morad F.P., Brunaldi K., Anania G., Pavanelli L., Pasqualucci A., Donini A. (2002). A new method of preemptive analgesia in laparoscopic cholecystectomy. Surg. Endosc. Other Interv. Tech..

[30] Bedreag O.H., Rogobete A.F., Sandesc D., Cradigati C.A., Sarandan M., Popovici S.E., Dumache R., Horhat F.G., Vernic C., Sima L.V. (2016). Modulation of the Redox Expression and Inflammation Response in the Crtically Ill Polytrauma Patient with Thoracic Injury. Statistical Correlations between Antioxidant Therapy and Clinical Aspects. Clin. Lab..

[31] David V.L., Ercisli F., Florin A., Boia E.S., Nitu R. (2017). Early Prediction of Sepsis Incidence in Critically Ill Patients Using Specific Genetic Polymorphisms. Biochem. Genet..

[32] Choi S., Yang S.Y., Choi G.J., Kim B.G., Kang H. (2019). Comparison of pressure- and volume-controlled ventilation during laparoscopic colectomy in patients with colorectal cancer. Sci. Rep..

[33] Hayden P., Cowman S. (2011). Anaesthesia for laparoscopic surgery. Contin. Educ. Anaesth. Crit. Care Pain.

[34] Berger M.M., Que Y.A. (2013). A protocol guided by transpulmonary thermodilution and lactate levels for resuscitation of patients with severe burns. Crit. Care.

[35] Küntscher M.V., Germann G., Hartmann B. (2006). Correlations between cardiac output, stroke volume, central venous pressure, intra-abdominal pressure and total circulating blood volume in resuscitation of major burns. Resuscitation.

[36] Li T.N., Li Y. (2014). Depth of anaesthesia monitors and the latest algorithms. Asian Pac. J. Trop. Med..

[37] Shah S., Chowdhury I., Bhargava A., Sabbharwal B. (2015). Comparison of hemodynamic effects of intravenous etomidate versus propofol during induction and intubation using entropy guided hypnosis levels. J. Anaesthesiol. Clin. Pharmacol..

[38] Riad W., Schreiber M., Saeed A.B. (2007). Monitoring with EEG entropy decreases propofol requirement and maintains cardiovascular stability during induction of anaesthesia in elderly patients. Eur. J. Anaesthesiol..

[39] Tewari S., Bhadoria P., Wadhawan S., Prasad S., Kohli A. (2016). Entropy vs. standard clinical monitoring using total intravenous anesthesia during transvaginal oocyte retrieval in patients for in vitro fertilization. J. Clin. Anesth..

[40] Wu S.C., Wang P.C., Liao W.T., Shih T.H., Chang K.A., Lin K.C., Chou A.K. (2008). Use of spectral entropy monitoring in reducing the quantity of sevoflurane as sole inhalational anesthetic and in decreasing the need for antihypertensive drugs in total knee replacement surgery. Acta Anaesthesiol. Taiwanica.

[41] Vakkuri A., Yli-hankala A., Sandin R., Mustola S. (2005). Spectral Entropy Monitoring Is Associated with Reduced Propofol Use and Faster Emergence in Propofol–Nitrous Oxide–Alfentanil Anesthesia. Anesthesiol. J. Am. Soc. Anesthesiol..

[42] El Hor T., Van Der Linden P., De Hert S., Melot C., Bidgoli J. (2013). Impact of entropy monitoring on volatile anesthetic uptake. Anesthesiology.

[43] Voss L., Sleigh J. (2007). Monitoring consciousness: The current status of EEG-based depth of anaesthesia monitors. Best Pract. Res. Clin. Anaesthesiol..

[44] Zhang Y., Shan G.J., Zhang Y.X., Cao S.J., Zhu S.N., Li H.J., Ma D., Wang D.X. (2018). Propofol compared with sevoflurane general anaesthesia is associated with decreased delayed neurocognitive recovery in older adults. Br. J. Anaesth..

[45] Kadoi Y., Kawauchi C., Saito S., Takahashi K. (2009). The comparative effects of equipotent Bispectral Index dosages of propofol and sevoflurane on cerebrovascular carbon dioxide reactivity in elderly patients. J. Clin. Anesth..

[46] Radtke F.M., Franck M., Lendner J., Krüger S., Wernecke K.D., Spies C.D. (2013). Monitoring depth of anaesthesia in a randomized trial decreases the rate of postoperative delirium but not postoperative cognitive dysfunction. Br. J. Anaesth..

[47] Llanes-garza H.A., López-cabrera N.G., Vega R.C., Palacios-rios D. (2015). Efficacy of antiemetic therapy in patients undergoing laparoscopic cholecystectomy. Med. Univ..

[48] Bedreag O.H., Papurica M., Rogobete A.F., Sarandan M., Cradigati C.A., Vernic C., Dumbuleu C.M., Nartita R., Sandesc D. (2016). New perspectives of volemic resuscitation in polytrauma patients: A review. Burn. Trauma.

[49] Bedreag O.H., Rogobete A.F., Sarandan M., Cradigati A.C., Papurica M., Dumbuleu M.C., Chira A.M., Rosu O.M., Sandesc D. (2015). Oxidative stress in severe pulmonary trauma in critical ill patients. Antioxidant therapy in patients with multiple trauma—A review. Anaesthesiol. Intensive Ther..

[50] Papurica M., Rogobete A.F., Sandesc D., Cradigati C.A., Sarandan M., Crisan D.C., Horhat F.G., Boruga O., Dumache R., Nilima K.R. (2016). The Expression of Nuclear Transcription Factor Kappa B (NF-kappaB) in the Case of Critically Ill Polytrauma Patients with Sepsis and Its Interactions with microRNAs. Biochem. Genet..

[51] Horhat F.G., Gundogdu F., David L.V., Boia E.S., Pirtea L., Horhat R., Cucui-Cozma A., Ciuca I., Diaconu M., Nitu R. (2017). Early Evaluation and Monitoring of Critical Patients with Acute Respiratory Distress Syndrome (ARDS) Using Specific Genetic Polymorphisms. Biochem. Genet..

[52] Dinu A.R., Rogobete A.F., Bratu T., Popovici S.E., Bedreag O.H., Papurica M., Bratu L.M., Sandesc D. (2020). Cannabis Sativa Revisited—Crosstalk between microRNA Expression, Inflammation, Oxidative Stress, and Endocannabinoid Response System in Critically Ill Patients with Sepsis. Cells..

[53] Longo M.A., Cavalheiro B.T., Filho G.R.D.O. (2017). Laparoscopic cholecystectomy under neuraxial anesthesia compared with general anesthesia: Systematic review and meta-analyses. J. Clin. Anesth..

[54] Fleming N., Cockerham R. (2019). General anaesthesia for operative obstetrics. Anaesth. Intensive Care Med..

[55] Cumpanas A.A., Bardan R., Ferician O.C., Latcu S.C., Duta C. (2017). Does previous open surgical experience have any influence on robotic surgery simulation exercises?. Videosurg. Other Miniinvasive Tech..

[56] Vãrcuæ F., Duåã C., Dobrescu A., Lazãr F., Papurica M., Tarta C. (2016). Laparoscopic Repair of Inguinal Hernia TEP versus TAPP. Chirurgia.

[57] Papurica M., Rogobete A.F., Sandesc D., Dumache R., Nartita R., Sarandan M., Cradigati A.C., Luca L., Vernic C., Bedreag O.H. (2015). Redox Changes Induced by General Anesthesia in Critically Ill Patients with Multiple Traumas. Mol. Biol. Int..

[58] Davidson A.J., Huang G.H., Rebmann C.S., Ellery C. (2005). Performance of entropy and Bispectral Index as measures of anaesthesia effect in children of different ages. Br. J. Anaesth..

[59] Zgaia A.O., Irimie A., Sandesc D., Vlad C., Lisencu C., Rogobete A., Achimas-Cadariu P. (2015). The role of Ketamine in the treatment of chronic cancer pain. Clujul Med..

[60] Hans P., Dewandre P., Brichant J.F., Bonhomme V. (2005). Comparative effects of ketamine on Bispectral Index and spectral entropy of the electroencephalogram under sevoflurane anaesthesia. Br. J. Anaesth..

[61] Brown A.C., Canosa-Mas C.E., Parr A.D., Pierce J.M.T., Wayne R.P. (1989). Tropospheric lifetimes of halogenated anaesthetics. Nature.

[62] Yasny J.S., White J. (2012). Environmental implications of anesthetic gases. Anesth. Prog..

